# Bidirectional associations between PTSD symptoms and pain in daily life among women survivors of sexual assault

**DOI:** 10.1016/j.janxdis.2025.103051

**Published:** 2025-07-08

**Authors:** Alexandra N. Brockdorf, Lauren E. Simpson, David DiLillo

**Affiliations:** a Medical University of South Carolina, United States; b University of Nebraska–Lincoln, United States

**Keywords:** Posttraumatic stress disorder, Pain, Sexual assault, Ecological momentary assessment, Comorbidity

## Abstract

The co-occurrence between pain and posttraumatic stress disorder (PTSD) is commonly explained by the mutual maintenance model, which proposes that each condition exacerbates the other. We tested this model by examining within-day associations between pain and PTSD using a three-week ecological momentary assessment (EMA) design. Young adult cisgender women (*N* = 82) who experienced sexual assault and reported PTSD symptoms and probable alcohol misuse completed three self-report surveys per day assessing momentary pain intensity and PTSD symptoms. Results from a dynamic structural equation model supported hypotheses, such that pain predicted greater PTSD symptoms four hours later and PTSD symptoms predicted greater pain. However, exploratory follow up analyses revealed differential findings by cluster, such that intrusions, negative alterations in cognition and mood, and hyperarousal each predicted subsequent pain, whereas pain predicted only later hyperarousal. Findings add nuance to our understanding of the mutual maintenance model and point to hyperarousal symptoms as a key symptom cluster linking daily pain and PTSD among women who have experienced sexual assault. Findings underscore the potential value of targeting hyperarousal symptoms in integrative interventions.

Pain is an unpleasant sensory experience that is either related to or resembles actual or potential tissue damage (Raja et al., 2020). Acute pain is an adaptive survival response to injury or tissue damage, acting as a protective signal to alert the body to danger. Chronic pain develops when pain persists beyond the healing period, losing its adaptive function, and is generally defined as lasting longer than three months (Cohen et al., 2021). Both types of pain are highly prevalent among sexual assault survivors. Sexual assault has been associated with pain in multiple body regions, including sites of direct injury and areas not physically harmed (McLean et al., 2012), as well as widespread pain (Ravyts et al., 2024). In a study of 549 women receiving care at sexual assault treatment centers, 72 % reported moderate to severe pain immediately after the assault, with 76 % experiencing clinically significant new or worsening pain one week later (Short et al., 2019). In another study, pain persisted or worsened three months after sexual assault among 60 % of women survivors (Ulirsch et al., 2014). These findings demonstrate that sexual assault survivors frequently experience new or worsening pain shortly after the assault, which can persist or worsen over time.

Posttraumatic stress disorder (PTSD) is one factor that may contribute to the persistence of pain among sexual assault survivors. PTSD involves four primary symptom clusters: intrusions (e.g., unwanted and distressing memories and dreams), avoidance of trauma-related stimuli, negative alterations in cognition and mood (e.g., distorted cognitions including self-blame, dampened positive emotions), and heightened arousal and reactivity (American Psychiatric Association, 2013). Like pain, PTSD is highly prevalent in this population, with sexual assault conferring the highest risk for developing PTSD compared to other trauma types (Brewin et al., 2025; Kessler et al., 2017). A recent meta-analysis found that 75 % of survivors met diagnostic criteria for PTSD one month after the assault, and 41 % met criteria one year later (Dworkin et al., 2023). PTSD and pain also share common vulnerabilities, such as anxiety sensitivity, which contribute to their co-occurrence and mutual maintenance (Asmundson et al., 2002). Sexual assault may uniquely impact both conditions, as it has been linked to specific types of pain (e.g., pelvic, genital, menstrual pain, Jina & Thomas, 2013), widespread pain (Ravyts et al., 2024), and heightened risk of developing PTSD (Kessler et al., 2017).

Not surprisingly given their high prevalence, PTSD and pain commonly co-occur, with research indicating high rates of both conditions (Pacella et al., 2013). Sharp and Harvey’s (2001) mutual maintenance model offers a framework for understanding this relation, proposing that PTSD and pain are reciprocally reinforcing. According to this model, trauma-related reminders, maladaptive cognitions, and heightened arousal can activate the stress response system and increase attentional focus on threat, thereby intensifying the experience of pain (Mickleborough et al., 2011; Timmers et al., 2019). Intrusions and flashbacks can also include vivid sensory elements, such as pain experienced during the assault (Brewin et al., 2025). Simultaneously, pain sensations may cue trauma memories, negative affect, and subsequent efforts to avoid stimuli evoking pain and trauma-related reminders (Frumkin & Rodebaugh, 2021; Lam et al., 2024). Over time, these associations may become paired where PTSD symptoms elicit conditioned pain responses (e.g., pain-related worry) and vice versa (Franke et al., 2022). Thus, PTSD symptoms may not only exacerbate acute pain following sexual assault but also contribute to the progression into chronic pain.

To date, the mutual maintenance model has been examined primarily using traditional longitudinal studies, with intervals for assessing pain and PTSD ranging from three weeks to six months. For example, among women who recently experienced sexual assault and sought emergency care, hyperarousal symptoms one week post-assault mediated the progression from acute to persistent pain over a period of six weeks (Short et al., 2022). Other longitudinal findings suggest that PTSD treatment reduces pain interference (i.e., the extent to which pain impedes functioning) and that pain interference is associated with greater PTSD symptom improvement over a three week period (Kovacevic et al., 2024). Findings from studies with longer follow-up periods, though less consistent, also largely support the mutual maintenance model. For instance, among military veterans, general pain symptoms predicted PTSD at six-month follow-up but not a year later, whereas PTSD predicted pain at both timepoints (Stratton et al., 2014). Conversely, in another study of military veterans, PTSD predicted pain interference three months later but not six months later, whereas pain predicted PTSD at both timepoints (Lee et al., 2019).

Despite this initial support for the mutual maintenance model, traditional longitudinal designs, with weeks or months between assessments, may miss the dynamic, bidirectional interactions between pain and PTSD symptoms that play out on a more short-term basis. Indeed, the intensity of chronic pain (Darnall & Sazie, 2012) and PTSD symptoms (Stefanovic et al., 2024) can fluctuate significantly within a day, highlighting a potential dynamic interplay where one symptom set directly influences the other over brief periods of time. Take, for example, a sexual assault survivor who experiences a spike in chronic pelvic pain (a common concern for survivors; Hassam et al., 2020) early in the morning. This increased pain could elicit intrusive memories of sexual assault and activate a physiological stress response leading to hyperarousal, which in turn may amplify the perception of pain later in the day (Short et al., 2022). This potential cycle of pain and PTSD symptoms—playing out in a matter of hours—is central to the mutual maintenance model, yet cannot be captured using traditional longitudinal assessments that aggregate data over time and thus lack the sensitivity to detect intra-day fluctuations.

By contrast, intensive longitudinal designs such as ecological momentary assessment (EMA) are well-suited for examining the short-term, reciprocal influences between pain and PTSD symptoms as they unfold in daily life. For instance, an EMA study among individuals who recently experienced motor vehicle accidents found that neck pain and traumatic stress symptoms covaried across the day, with pain predicting traumatic stress symptoms an hour later (Eather et al., 2019). Although traumatic stress symptoms did not predict future pain in contrast to the mutual maintenance model, findings speak to the proximal relations between PTSD symptoms and pain. In addition to capturing within-person variability in real time, EMA also reduces retrospective and recall biases by asking participants to report over shorter periods of time (e.g., Schuler et al., 2021). In the present study, we used EMA to examine whether PTSD exacerbates pain, whether pain worsens PTSD symptoms, or both, in the short term among women who have experienced sexual assault. In doing so, this study builds on prior longitudinal work and provides a more granular account of reciprocal relations between PTSD symptoms and pain, which could inform more targeted and effective interventions for sexual assault survivors experiencing both conditions.

Based on prior theory and empirical work supporting the mutual maintenance model, we hypothesized that, at the within-person level, greater PTSD symptoms would predict greater pain intensity several hours later, controlling for concurrent pain intensity and time in the study. Conversely, we expected that greater pain intensity would predict greater PTSD symptoms several hours later, controlling for concurrent PTSD symptoms and time in the study. To explore the possibility that specific PTSD symptom clusters might underlie the association between PTSD symptoms and pain (e.g., Short et al., 2022), we conducted exploratory analyses examining bidirectional associations between each PTSD symptom cluster and pain.

## 1. Method

### 1.1. Participants

Participants were recruited from a Midwestern city in the United States for a larger study of women who have experienced sexual assault that was advertised as examining activity levels and health behaviors in daily life (Brockdorf et al., 2024). Due to the larger study’s focus on drinking behavior among women who have experienced sexual assault, participants were required to identify as women, be between the ages of 18–30, own a cell phone, not be currently or planning to become pregnant, have experienced sexual assault since the age of 14 (i.e., endorsement of at least one of 14 behaviorally specific items indicating unwanted/nonconsensual oral sex or vaginal or anal penetration; Messman-Moore et al., 2010), report associated posttraumatic stress symptoms (i.e., score of six or more on the PTSD Symptom Checklist for DSM-5; Blevins et al., 2015), and report drinking at potentially hazardous levels (i.e., score of three or more on the Alcohol Use Disorders Identification Test - Consumption questionnaire; Bush et al., 1998). Over half of participants (57.3 %) experienced pain or were injured after sexual assault. Cisgender and transgender women were eligible to participate, but all participants identified as cis women. The mean age of participants was 22.8 (*SD* = 3.2). Approximately half (45.1 %) were full-time college students, 35.4 % were not students, 15.9 % were students in a graduate, technical, or professional school, and 2.4 % were part-time students. Most participants (73.2 %) were non-Hispanic White, followed by 13.4 % Hispanic/Latina, 9.8 % Black/African American, 6.1 % biracial or multiracial, 4.9 % American Indian/Alaska Native/Indigenous/Native American, 2.4 % Asian, and 2.4 % Middle Eastern/Arab (participants could endorse multiple categories so percentages exceed 100 %). Finally, 56.1 % of participants identified as heterosexual, 30.5 % as bisexual, 4.9 % as queer, 3.7 % as lesbian, 3.7 % as pansexual, and 1.2 % were unsure.

### 1.2. Measures

#### 1.2.1. PTSD symptoms

Momentary PTSD symptoms were assessed using an abbreviated version of the PTSD Symptom Checklist for DSM-5 (PCL-5; Blevins et al., 2015) that was modified for EMA use (Cox et al., 2025; Short et al., 2017). Ten items from the full PCL-5 were selected based on the strength of item loadings on their symptom cluster from the original validation study (Blevins et al., 2015): intrusions (unwanted memories, cued psychological distress, cued physical reactions), avoidance (internal avoidance, external avoidance), negative alterations in cognition and mood (social detachment, difficulty experiencing positive emotions), and hyperarousal (hyperarousal, feeling jumpy, difficulty concentrating). The response scale from the PCL-5 was not changed and ranged from 0 (*not at all*) to 4 (*extremely*). Instructions were modified such that participants reported the degree to which they were experiencing ten PTSD symptoms right now. For our primary outcome variable, all ten items were summed with a possible range of 0–40, with higher scores indicating greater momentary PTSD symptoms. This abbreviated version of the PCL-5 demonstrated high between-person level reliability (Ω =.93) and within-person level internal reliability (Ω =.82) in the current study. Further, recent psychometric evaluation of the abbreviated PCL-5 demonstrated high between-person (Ω =.96) and within-person level internal reliability (Ω =.82), as well as construct and convergent validity among sexual assault survivors (Cox et al., 2025). In addition to the total severity score, four symptom cluster subscales were created by computing the mean of the items that map onto each cluster as noted above. All four subscales demonstrated high between-person level internal reliability (Ω =.88–.99) but lower within-person reliability (Ω =.49–.78).

#### 1.2.2. Pain intensity

Momentary pain intensity was assessed using a single item: “How would you rate your physical pain right now?” (Hawker et al., 2011). Participants rated their pain on a visual analog scale ranging from 0 (*no pain*) to 100 (*worst imaginable pain)*. The pain visual analog scale is sensitive to change and demonstrates convergent validity with other measures of pain (Hawker et al., 2011; Kim & Jung, 2020). Single item measures are the most common method for assessing pain intensity in EMA surveys (May et al., 2018).

### 1.3. Procedures

All procedures were approved by the university’s IRB. Potential participants were recruited through local flyers and online advertisements that contained a link to an online informed consent and eligibility screening. Eligible participants then completed online baseline questionnaires and a brief in-person appointment to receive instructions for completing procedures during a three-week monitoring period. During the EMA phase, participants wore a wearable device assessing their sleep (beyond the scope of the present study) and completed three EMA surveys each day. Participants completed surveys using REDCap with Twilio integration to send survey links via text message (Harris et al., 2009). A stratified random assessment schedule was used to capture subjective experiences and behaviors dynamically as they unfold throughout the day, such that EMA surveys were randomly sent within the following set intervals: between 9:00 and 10:00 AM (morning), 1:00 and 2:00 PM (afternoon), and 5:00 and 6:00 PM (evening). PTSD symptoms and pain intensity were assessed at each of the three daily timepoints. At the end of the three weeks, participants completed a second in-person appointment to undergo debriefing and receive compensation. Participants could earn up to $166 for their participation.

### 1.4. Data analysis

Data were cleaned in SPSS Version 29 and all analyses were conducted in Mplus Version 8.11 (Muthén & Muthén, 1998). Internal reliability for composite measures was examined by computing within- and between-level Ω’s in Mplus. Intraclass correlations (ICCs) and participant-level averages were examined to describe the sample. To test the primary hypotheses, a dynamic structural equation model (DSEM) was estimated. We selected DSEM for the ability to accommodate a high number of observations per person, include lagged effects and account for unequal time intervals, simultaneously estimate PTSD symptoms and pain as dependent variables, and disaggregate within- and between-person level variance with latent mean centering. The Markov chain Monte Carlo (MCMC) procedure (i.e., the “Bayesian estimator”) was used with default noninformative priors, two MCMC chains, and the Gibbs algorithm. The default potential scale reduction (PSR) convergence criterion of nearing 1.00 was used. After initial convergence, the number of iterations was doubled as an additional check to make sure the PSR value stayed near 1.00. For each path, 95 % credibility intervals (CI) based on posterior distributions were examined and the effect was considered significant if the CI did not contain zero.

Our primary model examined reciprocal associations between PTSD symptoms and pain intensity (Level 1 *N* = 4582 EMA surveys). As standard in DSEM, PTSD symptoms and pain were disaggregated using latent mean centering. Hypotheses were tested at the within-person level, which represents fluctuations around each participant’s mean. At the within-person level, autoregressive paths (i.e., PTSD symptoms predicting PTSD symptoms at the next timepoint, pain predicting pain at the next timepoint), cross-lagged paths (i.e., PTSD symptoms predicting pain at the next timepoint, pain predicting PTSD symptoms at the next timepoint), and the concurrent association between PTSD symptoms and pain were estimated. To represent time in the study and account for potential reactivity, the ordered EMA survey number (i.e., running count from the first survey = 0 to the last survey = 62) was covaried with PTSD symptoms and pain at the within-person level. The number of EMA surveys was group-mean centered to aid interpretability. Regression slopes were fixed to improve the likelihood of convergence. The TINTERVAL command was used to account for unequal intervals between observations, which adds missing values to make intervals approximately evenly spaced (Hamaker et al., 2018). Because our assessment intervals were approximately four hours apart, we selected an interval of four hours. In addition to our primary model, we also conducted four supplemental models to explore reciprocal associations between pain and each of the four PTSD symptoms clusters. These models were estimated using the same procedures and were exploratory in nature.

## 2. Results

### 2.1. Descriptive statistics

Participants completed 88.7 % of all possible surveys (4582 of 5166 possible). ICCs indicated that 63.5 % of the total variance in PTSD symptoms was at the between-person level and 57.0 % of the variance in pain was at the between-person level. On average, participants reported a mean score of 3.57 on the abbreviated PCL-5 (ranged from 0 to 34) and a mean pain intensity of 19.32 (ranged from 0 to 100), indicative of low average PTSD symptoms and pain intensity. These low rates of momentary PTSD symptoms were surprising as participants reported an average score of 31.77 (*SD* = 17.67) on the full 20-item PCL-5 administered during the eligibility screening, which represents that the average participant was experiencing symptoms of PTSD around the threshold for clinical significance.

### 2.2. Dynamic structural equation models

Our primary DSEM results can be found in Table 1. At the within-person level, greater than usual pain intensity at a given timepoint (i. e., elevations above the person mean) significantly predicted greater pain intensity four hours later and greater than usual PTSD symptoms at a given timepoint significantly predicted greater PTSD symptoms four hours later. Supporting our bidirectional hypotheses, greater than usual pain intensity at a given timepoint significantly predicted greater PTSD symptoms four hours later and greater than usual PTSD symptoms at a given timepoint significantly predicted greater pain intensity four hours later. Both paths were significant after controlling for the autoregressive association and time in the study. Pain intensity and PTSD symptoms were significantly associated at a given timepoint. Greater time in the study was associated with lower PTSD symptoms but not significantly associated with pain intensity.

Regarding our exploratory analyses examining each PTSD symptom cluster (Table 2), greater than usual pain intensity at a given timepoint significantly predicted greater pain intensity four hours later and greater than usual PTSD symptoms in each cluster at a given timepoint significantly predicted greater PTSD symptoms in that same cluster four hours later across all models at the within-person level. Greater than usual pain intensity at a given timepoint significantly predicted greater hyperarousal symptoms four hours later, but not intrusions, avoidance, or negative alterations in cognition and mood. Greater than usual intrusions, negative alterations in cognition and mood, and hyperarousal symptoms (but not avoidance) at a given timepoint each significantly predicted greater pain intensity four hours later. Pain intensity at a given timepoint was associated with intrusions, avoidance, and hyperarousal at the same timepoint, but not negative alterations in cognition and mood. Greater time in the study was associated with lower avoidance and hyperarousal symptoms but was unrelated to intrusions or negative alterations in cognition and mood. In contrast, greater time in the study was associated with lower pain intensity in the models examining intrusions, avoidance, or negative alterations in cognition and mood, but not the model examining hyperarousal.

## 3. Discussion

The present study used EMA to provide a novel, proximal test of the mutual maintenance model of pain and PTSD (Sharp & Harvey, 2001) among women who have experienced sexual assault. As expected, greater than usual PTSD symptoms and pain intensity each predicted increases in the other four hours later. Exploratory analyses revealed differential associations between pain and each PTSD symptom cluster, with PTSD symptom clusters more consistently predicting subsequent pain than vice versa; pain predicted only later hyperarousal symptoms. These findings support the mutual maintenance model overall and highlight hyperarousal as a potentially key symptom cluster in the dynamic interplay between PTSD and pain following sexual assault.

These findings demonstrate within-person, bidirectional associations between global PTSD symptoms and pain, extending prior longitudinal research showing reciprocal effects over periods of months (Ravn et al., 2018). Our results indicate that such bidirectional associations unfold over much shorter time frames, drawing attention to a more proximal cycle where pain and PTSD symptoms may escalate within a single day. Specifically, elevations in either PTSD symptoms or pain predicted increases in the other several hours later. As posited by the mutual maintenance model, pain may act as a trauma reminder for women who have experienced sexual assault, worsening PTSD symptoms in the short term. In turn, PTSD symptoms may contribute to heightened focus on the body (e.g., physiological reactions to intrusions, feeling tense), reduced activity (e.g., avoiding social events, pleasurable activities), and diminished attentional control (e.g., due to the cognitive load of avoiding trauma-related thoughts and feelings), thereby increasing sensitivity to pain cues. These within-day associations underscore the importance of examining PTSD and pain in the context of sexual assault, given the distinct ways sexual assault may influence the severity, duration, and interplay of both conditions (Jina & Thomas, 2013; Kessler et al., 2017). Although the observed effects were statistically significant, it is important to note that cross-lagged estimates were generally small after accounting for prior symptoms and time in the study.

Findings from our exploratory analyses examining daily associations between pain intensity and individual PTSD symptom clusters add important nuance. Elevated pain intensity predicted small but significant increases in later hyperarousal but was unrelated to later increases in intrusions, avoidance, or negative alterations in cognition and mood. It is possible that heightened muscle tension associated with pain may mimic or exacerbate physiological symptoms of hyperarousal such as feeling tense or on edge. More intense pain could also interfere with attentional capacity, leaving individuals less able to concentrate or more prone to being startled as their focus shifts to managing their pain. It is also possible that unexamined third variables underlie the observed associations between pain and PTSD symptoms. For example, heightened anxiety sensitivity could amplify attention to unpleasant physiological sensations and their perceived negative consequences (Asmundson et al., 2002). Regarding the lack of association between pain and the other PTSD symptom clusters, it may be that the psychological impact of pain occurs in the short term but is not sustained long enough to influence PTSD symptoms four hours later (Eccleston & Crombez, 1999). Indeed, our findings that greater than usual PTSD symptoms were associated with greater than usual pain at the same timepoint could reflect the close proximity between PTSD symptoms and pain in daily life. This finding held true for associations between pain and individual PTSD symptom clusters, except for negative alterations in cognition and mood, which were unrelated to pain at the same timepoint.

As with global PTSD symptoms, greater than usual intrusions, negative alterations in cognition and mood, and hyperarousal—but not avoidance symptoms—predicted greater pain four hours later. Symptoms such as cued psychological distress and physiological reactivity activate stress responses in the hypothalamic-pituitary-adrenal (HPA) axis and autonomic nervous system that can increase muscle tension, inflammation, and pain sensitivity (Borsook et al., 2012; Timmers et al., 2019), contributing to greater pain later in the day. By contrast, survivors who tend to avoid thoughts and feelings of sexual assault may be less aware of their internal states, including pain sensations. On the other hand, avoidance of external activities and situations could promote inactivity, making it unlikely that pain would change in the following hours. Although avoidance is often highly reinforcing in the short-term, it is notable that avoidance symptoms did not predict reduced pain four hours later, suggesting it was not an effective strategy for preventing pain altogether.

### 3.1. Limitations

Although the present study provides a rigorous, proximal test of the mutual maintenance model using EMA, several limitations warrant consideration. Our sample consisted of cisgender women who experienced sexual assault, which means our findings may not generalize to trauma survivors with other gender identities (i.e., cisgender and transgender men, transgender women, gender nonbinary individuals) or types of traumatic exposure. For instance, witnessed events that do not involve direct bodily harm may be less strongly associated with pain than assaults involving physical injury. Although more than half of participants experienced physical pain or injury following their sexual assault, it is unclear whether the momentary reports of pain reflected new or worsening pain directly related to the assault. Participants were also predominantly White, which may limit generalizability. Individuals from marginalized racial/ethnic groups may experience even greater rates of pain due to disparities in access to effective pain medicine and management (Shavers et al., 2010). In addition, all participants reported probable alcohol misuse, as required for the larger study. Because alcohol is often used for pain relief (Ditre et al., 2019), this may have led to a sample more likely to endorse chronic pain. It is also possible that acute alcohol use altered momentary perceptions of pain and PTSD symptoms due to its dose dependent analgesic effects (Zale et al., 2015).

Levels of pain and PTSD symptoms were low on average across all EMA surveys, likely due to recruitment of a community sample without criteria for chronic pain or PTSD diagnosis. The low PTSD symptom levels could reflect our assessment of current symptoms (“right now”) and the use of an abbreviated set of PTSD symptoms. Although this reduced the burden of completing multiple daily assessments, not all symptoms in each cluster were represented, which may have led to the lower reliability for the PTSD symptom clusters at the within-person level. Although others have used similar approaches (Possemato et al., 2012; Short et al., 2017), there is need for continued development of measures specifically for use in intensive longitudinal studies of PTSD (Cox et al., 2025). Lastly, our single-item pain intensity measure, while common in EMA studies and medical practice (May et al., 2018), did not capture pain location or interference.

### 3.2. Future research directions

Future studies should include more comprehensive assessments of pain, including measures of pain location, functional interference, and perceived efficacy in managing pain. It may be especially important to assess pain types that are prevalent among sexual assault survivors, such as pelvic pain (Jina & Thomas, 2013). Measures assessing potential mechanisms for the demonstrated bidirectional associations (e.g., anxiety sensitivity, cognitive coping, physiological stress responses) may also be helpful. Qualitative studies examining perceptions of pain in relation to sexual assault and PTSD symptoms could shed light on novel mechanisms for future testing in intensive longitudinal studies. Laboratory-based studies could further elucidate causal processes by examining whether experimental manipulations of pain (e.g., cold pressor tasks) or trauma-related symptoms (e.g., trauma analogues) influence the other construct. Lastly, future research should explore whether dynamic fluctuations in pain and PTSD symptoms ultimately contribute to other adverse consequences, such as lower pain self-efficacy (i.e., beliefs about being unable to cope with or control pain) or maladaptive strategies for managing both pain and PTSD symptoms, such as substance use. Women who have experienced sexual assault are 68 % more likely to have received opioid prescriptions compared to women without sexual assault histories (Austin & Short, 2020) and PTSD confers heightened risk for developing opioid use disorder (Hassan et al., 2017). Moreover, given alcohol’s analgesic properties at certain thresholds, survivors may drink to self-medicate for pain relief (Ditre et al., 2019) and psychological distress (Nelson & Fischer, 2021). These future directions may be especially relevant in light of the observed associations between PTSD symptoms and pain in this sample of women sexual assault survivors reporting alcohol misuse.

### 3.3. Clinical implications

Clinicians should consider educating survivors on the reinforcing nature of PTSD and pain as our findings highlight that elevations in one might be closely followed by elevations in the other. Sharing this information could help survivors recognize dynamic changes and use coping skills in the moment to interrupt the bidirectional and mutually reinforcing nature of pain and PTSD symptoms. Just-in-time adaptive interventions that help survivors cope with PTSD symptoms and pain in the moment, such as sharing cognitive coping strategies, personalized suggestions for valued activities, and brief relaxation exercises, could also be useful in disrupting mutual maintenance.

Given the close link between PTSD symptoms and pain, integrated or transdiagnostic treatments may also be warranted (see Wolf & Otis, 2018 for a review). Integrated approaches, such as combining cognitive processing therapy (CPT) with cognitive-behavioral therapy for chronic pain (CBT-CP), have shown initial acceptability (Otis et al., 2009). A web-based acceptance and commitment therapy (ACT) program incorporating acceptance and mindfulness skills, values identification and in-vivo exposures, and written exposure exercises led to clinically significant reductions in PTSD symptoms and pain interference among patients with comorbid chronic pain and PTSD (Åkerblom et al., 2024). Although continued evaluation is needed, these studies show initial promise for treating PTSD and pain simultaneously. Moreover, our findings that PTSD symptom clusters more robustly predicted later pain than the reverse suggest that existing evidence-based interventions for PTSD could also have beneficial downstream effects on pain. For instance, a three-week intensive CPT program that included mindfulness training and trauma-sensitive yoga led to small decreases in pain interference and large decreases in PTSD symptoms among military veterans (Kovacevic et al., 2024). Treatments that focus on fear extinction (Knowles & Tolin, 2022), such as prolonged exposure and written exposure therapy, may also be useful for treating women sexual assault survivors with comorbid PTSD and pain, especially given the robust nature of associations between hyperarousal symptoms and pain intensity in this study.

### 3.4. Conclusion

The present findings extend prior longitudinal results supporting the mutual maintenance model by indicating that bidirectional prospective associations between PTSD symptoms and pain also emerge more dynamically across several hours among women who have experienced sexual assault. Across PTSD symptom clusters, hyperarousal was most consistently associated with pain and may be an important mechanism of change. Researchers and clinicians should consider and address the intersection of pain and PTSD symptoms among women who have experienced sexual assault.

## Figures and Tables

**Table 1:** Primary dynamic structural equation model results.

Estimated Path	*b*	*SE*	Lower 95 % CI	Upper 95 % CI	*B*
Within-Person Level (*R*^2^ = 28.6 % for PTSD symptoms, 18.8 % for pain)					
Pain → Pain four hours later	0.43*	0.02	.393	.461	.43
Pain → PTSD symptoms four hours later	0.01*	0.00	.001	.016	.04
PTSD symptoms → PTSD symptoms four hours later	0.53*	0.02	.499	.558	.53
PTSD symptoms → Pain four hours later	0.18*	0.06	.054	.307	.04
Correlation between PTSD symptoms and pain at the same timepoint	2.00*	0.60	.803	3.189	.05
Correlation between PTSD symptoms and time in the study	−1.88*	0.86	−3.541	−.186	−.04
Correlation between pain and time in the study	−7.01	3.69	−14.307	0.236	−.03

Note.

* = significant effect based on the 95 % CI crossing zero.

**Table 2:** Exploratory dynamic structural equation model results.

Estimated Path	Intrusions	Avoidance	Negative Alterations in Cognition and Mood	Hyperarousal
Pain → Pain	0.43*, 0.02 (.395,.462),.43	0.43*, 0.02 (.398,.465),.43	0.43*, 0.02 (.393,.461),.43	0.43*, 0.02 (.394,.462),.43
Pain → PTSD cluster	0.00, 0.00 (.000,.002),.03	0.00, 0.00 (−.001,.002),.01	0.00, 0.00 (.000,.002),.04	0.002*, 0.00 (.001,.003),.05
PTSD cluster → PTSD cluster	0.49*, 0.02 (.460,.521),.49	0.40*, 0.02 (.366,.437),.40	0.44*, 0.02 (.403,.470),.44	0.45*, 0.02 (.417,.481),.45
PTSD cluster → Pain	1.27*, 0.60 (.119, 2.451),.03	0.12, 0.46 (−.768, 1.030),.00	1.65*, 0.47 (.730, 2.592),.06	1.44*, 0.50 (.442, 2.430),.05
Correlation between PTSD cluster and pain	0.14*, 0.07 (.010,.279),.03	0.26*, 0.10 (.075,.448),.04	0.03, 0.09 (−.151,.214),.01	0.35*, 0.08 (.191,.512),.07
Correlation between PTSD cluster and time in the study	−0.13, 0.09 (−.310,.058), −.02	−0.28*, 0.13 (−.531, −.028), −.03	−0.11, 0.13 (−.350,.146), −.01	−0.49*, 0.11 (−.709, −.263),
Correlation between pain and time in the study	7.73*, 3.64 (−14.890, −.532),.03	8.23*, 3.64 (−15.360, −.997),.04	−7.49*, 3.63 (−14.644, −.342), −.03	−6.73, 3.66 (−13.833,.299), −.03
*R*^*2*^ for pain	18.8 %	18.7 %	18.9 %	19.0 %
*R*^*2*^ for PTSD cluster	24.5 %	16.3 %	19.5 %	21.2 %

Note.

* = significant effect based on the 95 % CI crossing zero. Unstandardized estimates, standard errors, 95 % credibility intervals, and standardized estimates are displayed.
